# Clinicopathological features and recurrence patterns of combined hepatocellular-cholangiocarcinoma

**DOI:** 10.1186/s12957-020-02099-w

**Published:** 2020-12-04

**Authors:** Takamichi Ishii, Takashi Ito, Shinji Sumiyoshi, Satoshi Ogiso, Ken Fukumitsu, Satoru Seo, Kojiro Taura, Shinji Uemoto

**Affiliations:** 1 Department of Surgery, Graduate School of Medicine, Kyoto University, 54 Kawahara-cho Shogoin, Sakyo-ku, Kyoto, 606-8507 Japan; 2 Department of Diagnostic Pathology, Kyoto University Hospital, 54 Kawahara-cho Shogoin, Sakyo-ku, Kyoto, 606-8507 Japan

**Keywords:** cHCC-CC, CHC, Recurrence

## Abstract

**Background:**

Combined hepatocellular-cholangiocarcinoma (cHCC-CCA) is a primary liver carcinoma with both hepatocellular carcinoma (HCC) and cholangiocarcinoma (CCA) components. We examined the clinicopathological characteristics and recurrence patterns of cHCC-CCA. Because of the rarity of cHCC-CCA, its etiology, clinicopathological features, and prognosis in comparison with other primary liver carcinoma remain unknown. Its recurrence pattern and sites in particular also need to be elucidated.

**Methods:**

All patients who underwent hepatectomy for primary liver malignancies between 2005 and 2015 were retrospectively included in this study.

**Results:**

Eight hundred and ninety-four hepatectomies were performed. Nineteen cases of cHCC-CCA (2.1%) in 16 patients were enrolled. Three patients underwent re-hepatectomy. The background of hepatitis viruses and tumor marker patterns of cHCC-CCA were similar to those of HCC and dissimilar to those of intrahepatic CCA (iCCA). Biliary invasion was common in cHCC-CCA and iCCA. The 5-year overall survival values of the cHCC-CCA, HCC, and iCCA patients were 44.7%, 56.6%, and 38.5%, respectively. The 5-year recurrence-free survival values of the cHCC-CCA, HCC, and iCCA patients were 12.2%, 28.7%, and 32.9%, respectively. The liver was the most common recurrence site. Unlike HCC, however, the lymph node was the second-most common recurrence site in both cHCC-CCA and iCCA. Pathological samples of the recurrent lesions were obtained in six patients, and four had cHCC-CCA recurrence pathologically.

**Conclusion:**

cHCC-CCA had a mixture of characteristics of HCC and iCCA. Many cases of cHCC-CCA remained cHCC-CCA pathologically even after recurrence.

## Introduction

Combined hepatocellular-cholangiocarcinoma (cHCC-CCA) is a relatively rare primary liver carcinoma that has both a hepatocellular carcinoma (HCC) component and cholangiocarcinoma (CCA) component in the same tumor nodule [1]. The classification of cHCC-CCA has been modified since Allen and Lisa first described it in 1949 [2], and the World Health Organization (WHO) recently updated its taxonomy [1, 3, 4]. Because of the rarity of cHCC-CCA, its etiology and clinicopathological features in comparison with other primary liver carcinoma remain unknown [5–11]. Although the prognosis of cHCC-CCA is often reported to be worse than that of HCC, the prognostic outcomes vary in comparison with that of iCCA [5, 8]. Its recurrence pattern and sites, the pathology of recurrent lesions, and the optimal treatment for recurrence in particular need to be elucidated [12].

In the present study, we examined the clinicopathological characteristics and recurrence patterns of cHCC-CCA patients who underwent hepatectomy in a single institution. We also compared cHCC-CCA with other the primary liver carcinomas, namely HCC and intrahepatic CCA (iCCA), to clarify the biological features of cHCC-CCA.

## Methods

### Study design

This study was a retrospective observational study in a single institution. The study protocol was approved by the Ethics Committee of the Graduate School of Medicine, Kyoto University (R1737), and performed in accordance with the 1964 Helsinki Declaration and its later amendments. Written informed consent was obtained from all study participants. All patients who received hepatectomy for primary liver malignancies between January 2005 and December 2015 were included in this study. Patients who underwent liver transplantation were excluded [13].

### Peri-operative management

All patients were evaluated preoperatively using chest X-ray and contrast-enhanced computed tomography (CT) of the chest and abdomen. Additional studies including magnetic resonance imaging (MRI) and positron emission tomography were performed as needed. Hepatic resection was performed as previously described [14–16]. In the case of HCC, anatomical resection is basically selected. However, non-anatomical resection may be selected in cases of patients with a poor liver function. Extrahepatic bile duct resection is usually avoided. Lymph node dissection is not performed. In cases of iCCA, anatomical resection is also selected in principle. If a tumor is suspected of having invaded the hilar bile duct, extrahepatic bile duct resection is performed. Lymph node dissection is basically performed; however, it may be omitted for peripheral iCCA [17]. All patients were followed up after surgery by serum tumor markers (alpha-fetoprotein (AFP), des-gamma-carboxy prothrombin (DCP), carcinoembryonic antigen (CEA), and carbohydrate antigen 19-9 (CA19-9)) and contrast-enhanced CT or MRI every 3 to 6 months. Recurrence was confirmed by imaging examinations, tumor markers, and pathological examinations [14].

### The pathological examination

A pathological examination was performed for all resected tumors and the background livers. The tumor size, tumor number, vascular invasion, serosal invasion, surgical margin invasion, and tumor differentiation were determined pathologically. The pathologic diagnosis was based on hematoxylin-eosin staining according to the WHO 2019 criteria [1], and immunohistochemical examinations for hepatocytic and cholangiocytic markers were added as needed to confirm the diagnosis.

This study defined major vascular invasion as tumor invasion to primary or secondary branches of the portal veins and/or biliary tract, and/or invasion to the main trunks of the hepatic veins or the inferior vena cava [14].

### Statistical analyses

Continuous data among three groups were analyzed using Tukey’s test. Categorical data among three groups were analyzed using the chi-square test. Survival curves were estimated by the Kaplan-Meier method and compared by the log-rank test with Bonferroni’s correction. A *P* value of < 0.05 was considered statistically significant. The JMP software package was used for all statistical analyses (JMP, Cary, NC).

## Results

### Clinicopathological features

From 2005 to 2015, a total of 894 cases underwent hepatectomy for primary liver tumors. Sixteen patients with cHCC-CCAs accounted for 19 cases that received hepatectomy (2.1%); 3 of the 16 cHCC-CCA patients underwent repeated hepatectomy (15.8%). Seven hundred and forty-two cases were HCCs, 121 cases were iCCAs, and 12 cases were other primary liver tumors, including malignant lymphoma, hepatic carcinosarcoma, and hepatic neuroendocrine tumor. One hundred and thirty-three of the 742 HCC patients (17.9%) and 11 of the 121 iCCA patients (9.0%) underwent repeated hepatectomy. During the same period of the study, three patients underwent living-donor liver transplantation for cHCC-CCA and were excluded from this study.

The clinicopathological features of cHCC-CCA, HCC, and iCCA are summarized in Table 1. HCC was more common in men than in women, and more patients had a worse liver function with HCC than those with iCCA. Although more than 90% patients with iCCA were negative for hepatitis B surface antigen (HBsAg), 15.8% of patients with cHCC-CCA and 19.8% of patients with HCC were positive for HBsAg. More than 80% patients with iCCA were negative for hepatitis C virus antibody (HCV-Ab), whereas more than 40% of patients with cHCC-CCA and HCC were positive for HCV-Ab. The preoperative CA19-9 levels in the iCCA patients were significantly higher than those in the cHCC-CCA or HCC patients. Patterns of viral markers and tumor markers in the cHCC-CCA patients more closely resembled those of HCC patients than those of iCCA patients. All patients with cHCC-CCA had a single cHCC-CCA tumor nodule, and 4 of the 16 cHCC-CCA patients had synchronous HCC tumor nodules. There were no significant differences in the rates of major portal vein or hepatic vein invasion among the three groups. However, the frequencies of major biliary tract invasion in cHCC-CCA and iCCA (36.8% and 18.2%, respectively) were higher than that in HCC (5.0%). At the site of the invasion into the major biliary tract of the seven cHCC-CCA patients, three had cHCC-CCA tumors, two had HCC tumors, one had an iCCA tumor, and one had an indeterminate pathology. The surgical procedures for cHCC-CCA, including the presence or absence of resection of extrahepatic bile duct and/or lymph node dissection, were similar to those for HCC, as the preoperative diagnosis of the cHCC-CCA patients was often HCC.

**Table 1:** The clinicopathological characteristics of the cHCC-CCA, HCC, and iCCA cases. *HBsAg* hepatitis B surface antigen, *HCV-Ab* hepatitis C virus antibody, *AFP* alpha-fetoprotein, *DCP* des-gamma-carboxy prothrombin, *CEA* carcinoembryonic antigen, *CA19-9* carbohydrate antigen 19-9, *AR* anatomical resection, *NAR* non-anatomical resection, *NS* not significant. Numbers are described as the mean ± standard deviation. Values in parentheses are percentages

	cHCC-CCA (***n*** = 19)	HCC (***n*** = 742)	iCCA (***n*** = 121)	Statistical analysis
**Sex**	Male: 12 Female: 7	Male: 583 Female: 159	Male: 72 Female: 49	*p* < 0.0001
**Age**	64.1 ± 10.6	66.7 ± 9.7	66.7 ± 10.0	NS
**Platelet number (× 10**^**4**^**/μL)**	17.2 ± 10.7	13.6 ± 7.8	19.1 ± 9.0	HCC vs. iCCA: *p* < 0.0001
**Total bilirubin (mg/dL)**	1.19 ± 0.71	1.05 ± 1.21	0.89 ± 0.72	NS
**Albumin (g/dL)**	3.91 ± 0.50	3.82 ± 0.54	3.99 ± 0.47	HCC vs. iCCA: *p* = 0.0032
**Prothrombin time (%)**	83.6 ± 12.0	83.7 ± 15.7	91.6 ± 16.0	HCC vs. iCCA: *p* < 0.0001
**HBsAg**	Positive: 3 (15.8%) Negative: 16 (84.2%) Missing data: 0	Positive: 147 (19.8%) Negative: 588 (79.2%) Missing data: 7	Positive: 7 (5.8%) Negative: 109 (90.1%) Missing data: 5	*p* < 0.0001
**HCV-Ab**	Positive: 8 (42.1%) Negative: 11 (57.9%) Missing data: 0	Positive: 336 (45.3%) Negative: 400 (53.9%) Missing data: 6	Positive: 16 (13.2%) Negative: 102 (84.3%) Missing data: 3	*p* < 0.0001
**AFP (ng/mL)**	110.7 ± 343.5	7416 ± 108118.1	12.6 ± 49.3	NS
**DCP (mAU/mL)**	1613.3 ± 5099.2	7895.5 ± 35673.2	693.1 ± 3890.6	NS
**CEA (ng/mL)**	2.88 ± 3.01	3.65 ± 2.61	7.36 ± 18.9	HCC vs. iCCA: *p* = 0.0015
**CA19-9 (U/mL)**	37.2 ± 31.6	48.3 ± 55.1	308.7 ± 760.4	HCC vs. iCCA: *p* < 0.0001 cHCC-CCA vs. iCCA: *p* = 0.0294
**Tumor number**	1.21 ± 0.42	1.68 ± 1.56	1.48 ± 1.70	NS
**Tumor size (cm)**	4.07 ± 2.39	4.68 ± 4.19	4.63 ± 2.75	NS
**Major portal vein invasion**	Presence: 3 (15.8%) Absence: 16 (84.2%) Missing data: 0	Presence: 67 (9.0%) Absence: 618 (83.3%) Missing data: 57	Presence: 14 (11.6%) Absence: 94 (77.7%) Missing data: 13	NS
**Major hepatic vein invasion**	Presence: 0 (0%) Absence: 19 (100%) Missing data: 0	Presence: 25 (3.4%) Absence: 658 (88.7%) Missing data: 59	Presence: 9 (7.4%) Absence: 99 (81.8%) Missing data: 13	NS
**Major biliary tract invasion**	Presence: 7 (36.8%) Absence: 12 (63.2%) Missing data: 0	Presence: 37 (5.0%) Absence: 646 (87.1%) Missing data: 59	Presence: 22 (18.2%) Absence: 80 (66.1%) Missing data: 19	*p* < 0.0001
**Surgical procedure**	AR: 13 (68.4%) NAR: 6 (31.6%)	AR: 471 (63.5%) NAR: 271 (36.5%)	AR: 95 (78.5%) NAR: 26 (21.5%)	*p* = 0.0053
**Resection of extrahepatic bile duct**	Presence: 1 (5.3%) Absence: 18 (94.7%)	Presence: 3 (0.4%) Absence: 739 (99.6%)	Presence: 29 (24.0%) Absence: 92 (76.0%)	*p* < 0.0001
**Lymph node dissection**	Presence: 1 (5.3%) Absence: 18 (94.7%)	Presence: 9 (1.2%) Absence: 733 (98.8%)	Presence: 93 (76.9%) Absence: 28 (23.1%)	*p* < 0.0001

### Prognosis

**Fig. 1:**
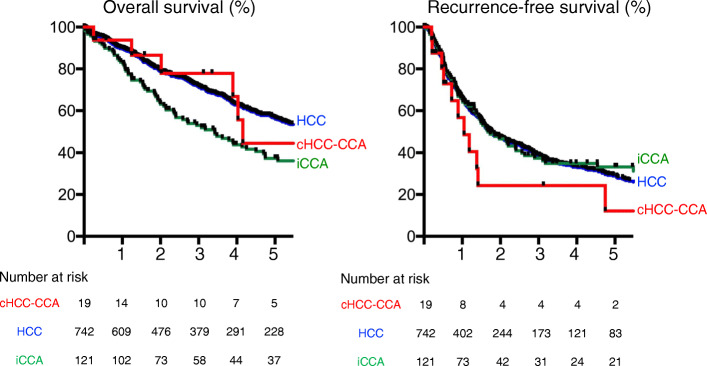
The left panel shows the Kaplan-Meier curve for the overall survival, and the right panel shows that for the recurrence-free survival between the patients with cHCC-CCA (red line), HCC (blue line), and iCCA (green line). The horizontal axis indicates the years after surgery

The overall survival (OS) was compared among cHCC-CCA, HCC, and iCCA patients (Fig. 1; left panel). The 5-year OS values of the cHCC-CCA, HCC, and iCCA patients were 44.7%, 56.6%, and 38.5%, respectively. The median survival times (MSTs) of the cHCC-CCA, HCC, and iCCA patients were 50.5, 72.2, and 41.7 months, respectively. The patients with iCCA had a worse prognosis than those with HCC with statistical significance (*p* = 0.0029). The recurrence-free survival (RFS) was also compared among the three groups (Fig. 1; right panel). The 5-year RFS values of the cHCC-CCA, HCC, and iCCA patients were 12.2%, 28.7%, and 32.9%, respectively. The median RFS values of the cHCC-CCA, HCC, and iCCA patients were 12.8, 21.3, and 20.3 months, respectively. There were no significant differences among the three groups.

### Treatment of recurrent cHCC-CCA

Tumor recurrences and/or metastases were confirmed using imaging examinations, such as CT or MRI, and occasionally pathological examinations. The liver was the most common site of tumor recurrence among the three tumor groups (Table 2). Although the lymph nodes were the second-most common site of recurrence in both cHCC-CCA and iCCA, lymph node metastasis was less frequent in HCC. Two of the three cHCC-CCA patients with lymph node recurrences had metastases in the hepatoduodenal ligament. The treatment modalities for recurrent cHCC-CCA are summarized in Table 3. The majority of the recurrent cHCC-CCA patients were treated according to HCC recurrence.

**Table 2:** The recurrence sites of cHCC, HCC, and iCCA. The values in parentheses are the percentages of all cHCC, HCC, and iCCA cases. In patients with multiple organ recurrence, each organ was counted

	cHCC-CCA	HCC	iCCA
**Liver**	11 (57.9%)	412 (55.5%)	48 (39.7%)
**Lung**	3 (15.8%)	83 (11.2%)	19 (15.7%)
**Lymph node**	3 (15.8%)	40 (5.4%)	21 (17.4%)
**Biliary tract**	1 (5.2%)	0	0
**Adrenal gland**	1 (5.2%)	14 (18.9%)	2 (1.7%)
**Bone**	1 (5.2%)	39 (5.3%)	16 (13.2%)
**Peritoneum**	0	10 (1.3%)	11 (9.1%)
**Brain**	0	11 (1.5%)	1 (0.8%)

**Table 3:** Therapeutic modalities for cHCC-CCA recurrence. *TACE* transarterial chemoembolization, *TAE* transarterial embolization, *RFA* radiofrequency ablation, *PEIT* percutaneous ethanol injection therapy, *HAIC* hepatic arterial infusion chemotherapy, *TKI* tyrosine-kinase inhibitor

**Surgery**	**8**
- Hepatic resection	7
- Non-hepatic resection	1
**TACE/TAE**	**4**
**RFA/PEIT**	**4**
**HAIC**	**1**
**Chemotherapy**	**4**
- TKI	3
- Gemcitabine	1
**Radiation**	**2**

### Pathological patterns of cHCC-CCA recurrence

Pathological examinations were performed in 6 patients, and 10 samples were obtained for recurrent and/or metastatic tumors. The pathological patterns and sites of cHCC-CCA recurrence in the six patients are summarized in Table 4. Four of the patients (B, C, D, and F) had recurrent tumors of cHCC-CCA. The main component of the primary tumor in patient A was CCA, and its lymph node recurrence was CCA. However, the main component of the primary tumor in patient E was HCC, and its subsequently repeated liver recurrences were HCC.

**Table 4:** The pathological patterns of cHCC-CCA recurrence in six patients. The letters in parentheses indicate the recurrent organs. *Ly* lymph node, *L* liver, *B* bile duct

	Recurrent pattern
**Patient A**	cHCC-CCA → CCA (Ly)
**Patient B**	cHCC-CCA → cHCC-CCA (L)
**Patient C**	cHCC-CCA → cHCC-CCA (L)
**Patient D**	cHCC-CCA → cHCC-CCA (L) → cHCC-CCA (Ly)
**Patient E**	cHCC-CCA → HCC (L) → HCC (L) → HCC (L)
**Patient F**	cHCC-CCA → HCC (L) → cHCC-CCA (B)

## Discussion

Combined HCC-CCA is a rare primary liver carcinoma, and 2.1% of the patients who underwent surgeries for primary liver carcinoma had cHCC-CCA in our hospital, which is similar to the incidences described in previous reports [5–12, 18, 19]. Due to the small number of cHCC-CCA cases, it was difficult to show a statistically significant difference in a comparison among three groups. However, many patients with cHCC-CCA were male, tended to be affected by hepatitis B virus and/or hepatitis C virus, and showed high AFP and/or DCP values and normal levels of CEA and CA19-9, which resembled the clinical features of HCC but not those of iCCA. However, the cHCC-CCA patients had a higher frequency of major biliary tract invasion than the HCC patients did, showing an affinity for the biliary tract. This characteristic was similar to that of iCCA. Although there was no statistical significance, the 5-year OS and RFS rates of cHCC-CCA fell between those of HCC and iCCA. Taken together, these clinical and prognostic features suggest that cHCC-CCA has mixed characteristics of HCC and iCCA.

Pathological samples for recurrent tumors were obtained from six cHCC-CCA patients. The pathological examinations revealed that four of the six patients had cHCC-CCA in their recurrent tumors. These findings suggested that cHCC-CCA belongs to a different disease category from HCC or iCCA. However, the majority of the recurrent cHCC-CCA patients were diagnosed without pathological examinations. Therefore, a more detailed pathological evaluation on cHCC-CCA recurrence should be performed in the future.

The liver was the most common site of recurrence in cHCC-CCA, as in HCC and iCCA. However, it was similar to iCCA in that there were many lung and/or lymph node metastases in cHCC-CCA. The majority of the non-resected cases were treated using the HCC treatment protocol. This was partially because the pattern of tumor markers and the imaging findings of cHCC-CCA recurrence resemble those of HCC. The 5-year OS of cHCC-CCA was higher than that of iCCA, although the 5-year RFS of cHCC-CCA was worse than that of iCCA. This was probably because cHCC-CCA was similar to HCC and had a better response to treatment for recurrence. Due to the small number of the cHCC-CCA cases, the optimal treatment for cHCC-CCA recurrence remains unclear [20–22]. However, to our knowledge, this report is the first to describe the characteristics of cHCC-CCA recurrence in detail [12, 23].

It is difficult to diagnose cHCC-CCA preoperatively because there are no imaging characteristics specific to cHCC-CCA [24, 25]. In the present study, all patients with cHCC-CCA were treated as having HCC; they received hepatectomy without lymph node dissection, and a pathological examination of the surgical specimens subsequently revealed cHCC-CCA. Given that the lymph nodes were the second-most common recurrence site in cHCC-CCA, hepatectomy with lymph node dissection might be necessary if a diagnosis of cHCC-CCA is made preoperatively [23, 26].

## Conclusion

cHCC-CCA had intermediate characteristics between HCC and iCCA in many aspects. Many cases of cHCC-CCA remained cHCC-CCA pathologically even after recurrence.

## Data Availability

The datasets used and analyzed during the current study are available from the corresponding author on reasonable request.

## Abbreviations

AFP: Alpha-fetoprotein
CCA: Cholangiocarcinoma
cHCC-CCA: Combined hepatocellular-cholangiocarcinoma
DCP: Des-gamma carboxyprothrombin
HCC: Hepatocellular carcinoma
iCCA: Intrahepatic-cholangiocarcinoma
